# Professional identity profiles among nursing interns: a latent profile analysis and associations with psychological capital

**DOI:** 10.3389/fpsyg.2026.1915176

**Published:** 2026-10-06

**Authors:** Shan Liu, Xu Guan, Juan Lian, Yisong Yao, Xiaomei Chen, Yuchen Fu, Yue Hao

**Affiliations:** 1 Nursing Department, The 3rd Affiliated Hospital of Chengdu Medical College, Chengdu Pidu District People's Hospital, Chengdu, China; 2 School of Health in Social Science, The University of Edinburgh, Edinburgh, United Kingdom; 3 The Primary Health Development Research Center of Sichuan Province, North Sichuan Medical College, Nanchong, China; 4 Nursing Department, The People's Hospital of Wenjiang Chengdu, Chengdu, China; 5 Department of Digestive Oncology, Shanxi Bethune Hospital, Shanxi Academy of Medical Sciences, Third Hospital of Shanxi Medical University, Tongji Shanxi Hospital, Taiyuan, China

**Keywords:** clinical education, latent profile analysis, nursing interns, professional identity, psychological capital

## Abstract

**Background:**

Clinical practicum is the stage at which nursing interns begin to experience nursing as daily work. Professional identity is important during this transition, yet interns are often treated as a homogeneous group because they share the same training stage. This may obscure different patterns of role understanding, clinical belonging, teamwork, coping, and reflection. This study aimed to identify latent profiles of professional identity among nursing interns and examine how dimensions of psychological capital were associated with profile membership.

**Methods:**

A cross-sectional survey was conducted in December 2024 in two tertiary general hospitals in Chengdu, China. Nursing interns were eligible if they had completed at least one month of clinical practicum. Professional identity was measured across five dimensions, and psychological capital was measured through self-efficacy, resilience, hope, and optimism. Latent profile analysis identified professional identity profiles. Profile differences were examined with appropriate group comparison tests, and multinomial logistic regression examined factors associated with profile membership.

**Results:**

Among 432 valid responses, a three-profile solution showed clear classification and meaningful class sizes. The low, moderate, and high professional identity profiles included 164, 192, and 76 nursing interns, and the estimated profile proportions were 38.5, 43.9, and 17.6%. Psychological capital scores increased across the three profiles. In multinomial logistic regression, self-efficacy was associated with membership in both the moderate profile (OR = 1.349, 95% CI = 1.195–1.523) and the high profile (OR = 1.412, 95% CI = 1.221–1.631), while optimism was also associated with the moderate profile (OR = 1.334, 95% CI = 1.051–1.693) and especially the high profile (OR = 2.204, 95% CI = 1.336–3.636). Hope was associated only with membership in the high profile (OR = 1.344, 95% CI = 1.103–1.636). Resilience was not independently associated with profile membership, and failed courses showed sparse-cell instability.

**Conclusion:**

Nursing interns at the same clinical practicum stage showed distinct professional identity profiles. Psychological capital, such as self-efficacy, optimism, and hope, was associated with membership in profiles characterized by stronger professional identity patterns. These findings may inform the design and future evaluation of profile-informed practicum support.

## Introduction

Nursing education is closely tied to the stability of the nursing workforce. Global nursing policy links education quality, employment conditions, and leadership with workforce capacity (World Health Organization, 2020; World Health Organization, 2021). Before professional registration, nursing students are already deciding whether they can see themselves as future nurses (Wang et al., 2022). This makes professional identity (PI) during clinical practicum an early workforce concern. PI refers to how much individuals identify with and accept the profession they have learned (Wang et al., 2022; Geoghan Marold et al., 2025). It is a process of gradually understanding and shifting into a positive state during learning, which is central to this judgement because it connects role understanding, professional values, belonging, and career intention (Geoghan Marold et al., 2025). Evidence from nursing workforce research indicates that PI is related to the choice to remain in nursing (Kristoffersen, 2021). PI during clinical practicum is therefore both an educational outcome and part of the preparation of a sustainable nursing workforce.

Clinical practicum is the point at which nursing students meet the profession as daily work (Liu et al., 2022). In China, nursing interns spend a substantial period in hospital departments before graduation, where they provide care under supervision and learn from preceptors, peers, patients, and clinical teams (Zhang et al., 2022). This setting gives interns direct access to professional role experience. It also exposes them to workload, feedback, uncertainty, and emotional pressure. Internship experience and career planning have been associated with PI among nursing students (Wei et al., 2021; Ma et al., 2022). Work atmosphere and teacher capacity have also been linked with PI among nursing interns (Zeng et al., 2022). Clinical learning environment may shape PI through career self-efficacy, which places confidence and learning support inside the identity formation process (Gao et al., 2022). These findings suggest that the same practicum stage can contain different identity experiences.

Professional Identity in Nursing helps explain why these experiences may differ across interns (Goodolf and Godfrey, 2021). PI develops through personal experience, education, professional socialization, and recognition from the practice environment (Mao et al., 2021). In clinical practicum, these broad processes become visible through specific dimensions. Professional cognitive evaluation concerns how interns understand nursing and its value. Professional social support concerns recognition from people around the intern. Professional social skills concern participation in patient care and teamwork. Professional frustration coping concerns responses to difficulty in clinical work. Professional self-reflection concerns the link between practicum experience and future career identity (Goodolf and Godfrey, 2021; Joseph et al., 2023). These dimensions make PI observable as a pattern of role understanding, belonging, participation, coping, and reflection. PI can also be understood as a developmental construct rather than a fixed attribute (Mao et al., 2021). During clinical practicum, nursing interns may move through different stages of role understanding, belonging, adaptation, and reflection (Goodolf and Godfrey, 2021; Mao et al., 2021). Even within the same training stage, interns may therefore occupy different positions in identity development. This developmental perspective supports a person-centered framework, because differences among interns may be expressed not only as higher or lower average scores but also as distinct patterns of PI across dimensions.

LPA provides a person-centered approach for identifying subgroups with similar response patterns across multiple indicators (Spurk et al., 2020). Emerging nursing research supports the importance of this perspective. PI profiles during clinical practicum have been associated with stress and coping styles (Li et al., 2023), and differences in PI have also been observed across self-directed learning profiles (Zhou et al., 2023). Resilience has further been examined as a psychological resource related to PI through personality-related pathways (Wu et al., 2024). Nevertheless, an important gap remains. Existing person-centered evidence has not adequately examined whether the four dimensions of psychological capital—self-efficacy, hope, resilience, and optimism—show distinct associations with PI profile membership among nursing interns in clinical practicum. This limits a more precise understanding of which modifiable psychological resources may be most relevant to targeted practicum support.

Psychological capital (PsyCap) provides a focused way to examine these resources. Psychological Capital Theory defines PsyCap as a developable positive psychological state that includes self-efficacy, hope, resilience, and optimism (Luthans et al., 2006). These components fit the demands of clinical practicum. Interns need confidence to perform clinical tasks, hope to set feasible goals, resilience to recover from difficulty, and optimism to interpret challenges as manageable. In nursing students, PsyCap has been related to perceived stress, self-directed learning ability, and PI (Feng et al., 2024). This evidence supports PsyCap as a relevant resource for understanding identity formation in nursing education. It also raises a more precise question for clinical practicum. Different PsyCap dimensions may relate to different PI profiles, and a total score alone may hide this pattern.

This study integrates Professional Identity in Nursing and Psychological Capital Theory within a person-centered analytic framework (Goodolf and Godfrey, 2021; Spurk et al., 2020; Luthans et al., 2006). It focuses on nursing interns during clinical practicum, a stage in which professional role experience and psychological resources meet in routine care settings. The study aimed to identify latent profiles of PI among nursing interns and to examine how PsyCap dimensions were associated with profile membership. Findings may help identify modifiable psychosocial resources for more targeted practicum support.

## Methods

### Study design and setting

This study was conducted in December 2024 in two tertiary general hospitals in Chengdu, China. It examined PI among nursing interns during clinical practicum and explored how PsyCap components were associated with latent PI profile membership. The clinical practicum setting was chosen because nursing interns are moving from classroom-based learning into direct clinical work, a period in which professional values, role understanding, and career commitment may become more visible (Zeng et al., 2022; Gong et al., 2025).

### Participants

The inclusion criteria were nursing interns who had completed at least one month of clinical practicum, understood the study information, and provided informed consent. Interns were excluded if they were absent from their clinical post during data collection because of sick leave, personal leave, or other reasons. The required sample size was estimated using Kendall’s rough rule for multivariate questionnaire studies, which recommends a sample size of 5 to 10 times the number of study variables (Kendall, 1980). The study involved 50 variables. A 20% invalid response allowance was added, giving a minimum required sample size of 300 participants. A total of 450 questionnaires were returned. After screening, 432 valid questionnaires were included in the analysis, giving a valid response rate of 96.0%.

### Measures

A demographic questionnaire was developed for this study after reviewing relevant literature and consulting experts in nursing education and clinical management. The questionnaire collected information on sex, age, educational level, the number of failed courses during school, and cumulative clinical practicum duration. These variables were selected because they reflect basic educational and clinical exposure characteristics that may relate to professional development during internship.

PI was measured with the Professional Identity Rating Scale developed by Liu et al. (2011). The scale contains 30 items across five dimensions: professional cognitive evaluation, professional social support, professional social skills, professional frustration coping, and professional self-reflection. Each item is rated on a 5-point Likert scale from 1, indicating strong disagreement, to 5, indicating strong agreement. Total scores range from 30 to 150. A higher score indicates a higher level of PI. The original scale showed good psychometric performance (Liu et al., 2011). In this study, the Cronbach’s alpha of this scale is 0.817. The raw sum score of each PI dimension was used as an indicator variable in the LPA.

PsyCap was measured with the Psychological Capital Scale developed by Luthans et al. (2006) and adapted into Chinese by Luo and He (2010). The scale contains 20 items across four dimensions: self-efficacy, resilience, hope, and optimism. Each item is rated on a 6-point Likert scale from 1, indicating strong disagreement, to 6, indicating strong agreement. Total scores range from 20 to 120. A higher score indicates a higher level of PsyCap. The Chinese version has been used among nursing populations in China and has shown good reliability (Luo and He, 2010). The four dimension scores were entered as continuous variables in the profile comparison and regression analyses. In this study, the Cronbach’s alpha of this scale is 0.793.

### Data collection

Data were collected through an online survey platform. The nursing departments of the two hospitals distributed the survey link to eligible nursing interns. The opening page explained the study purpose, participation requirements, confidentiality protection, and informed consent process. Participants completed the questionnaire anonymously after consent was provided.

Several steps were used to protect data quality. All items were set as mandatory in the online platform. Each IP address and each device could submit one response. Two researchers checked the returned questionnaires. Responses were excluded if completion time was <5 min or if answer patterns suggested careless completion, such as identical responses across a long series of items. The final analytic dataset included 432 valid questionnaires. The mandatory item setting meant that the final dataset contained no missing values for the variables used in the analysis.

### Statistical analysis

LPA was conducted with Mplus 8.7 to identify PI profiles among nursing interns (Muthen and Muthen, 2017). The five dimensions of the Professional Identity Rating Scale were used as indicator variables. Models with one to four profiles were fitted in sequence. Model fit was assessed with the Akaike information criterion (AIC), Bayesian information criterion (BIC), sample-size adjusted Bayesian information criterion (aBIC), entropy, Lo–Mendell–Rubin likelihood ratio test (LMR), and bootstrapped likelihood ratio test (BLRT) (Akaike, 1974; Schwarz, 1978; Sclove, 1987; Celeux and Soromenho, 1996; Lo et al., 2001; McLachlan, 1987). Lower information criterion values indicated better model fit. Entropy values close to 1 indicated clearer classification. Significant likelihood ratio tests indicated that a model with k profiles fitted the data better compared with a model with k-1 profiles. The final model was selected by combining statistical fit, classification accuracy, profile size, model parsimony, and substantive interpretability (Nylund et al., 2007).

Descriptive analyses and profile comparisons were performed with Statistical Package for the Social Sciences (SPSS) 27.0 (IBM Corp, 2020). Categorical variables were summarized as frequencies and percentages. Continuous variables were summarized with means and standard deviations (SDs). Demographic variables were compared across latent profiles using chi-square tests for nominal categorical variables and rank-based non-parametric tests for ordinal variables (Pearson, 1900; Kruskal and Wallis, 1952). Differences in PsyCap dimension scores across profiles were examined with analysis of variance (Fisher, 1925). Multinomial logistic regression was used to examine factors associated with PI profile membership (Theil, 1969). The low PI profile (C1) was used as the reference group. PsyCap dimensions and variables that differed across profiles in univariable analyses were entered as covariates in the multinomial logistic regression model. Odds ratios (ORs) and 95% confidence intervals (CIs) were reported (Neyman, 1937). Statistical significance was set at a two-sided *p* value <0.05 (Fisher, 1925).

### Ethical considerations

This study was approved by the Institutional Ethics Committee of Wenjiang District People’s Hospital (Approval No. 2023-008). Participation was voluntary. Informed consent was obtained before questionnaire completion. All questionnaires were anonymous.

## Results

### Participant characteristics

A total of 432 nursing interns were included in the analysis. The participants were aged 20–24 years, with a mean age of 21.04 years (SD = 1.76). Most participants were female (*n* = 358, 82.87%). Junior college programme students accounted for 63.89% of the sample, and bachelor’s degree programme students accounted for 36.11%. Most participants had no failed courses during school (*n* = 340, 78.70%). The cumulative clinical practicum duration was concentrated in the longer practicum groups, with 42.13% reporting 4–7 months and 52.31% reporting at least 8 months.

### LPA model selection

LPA was conducted using the five dimensions of PI as indicator variables. Table 1 shows the fit indices for the LPA models. The three-profile model showed strong classification quality, with AIC = 10017.494, BIC = 10106.999, aBIC = 10037.184, entropy = 0.959, LMR *p* = 0.004 and BLRT *p* < 0.001. The four-profile model created a very small class (0.93%). The three-profile model was retained because it gave clear class sizes and clinically meaningful profiles.

**Table 1:** Fit indices for latent profile models of professional identity.

Profile	Information criteria			Classification	Likelihood ratio tests		Profile proportions (%)
	AIC^a^	BIC^b^	aBIC^c^	Entropy	LMR^d^ P^e^	BLRT^f^ P	
1	12333.836	12374.520	12342.786	—	—	—	—
2	10970.205	11035.300	10984.525	0.923	<0.001	<0.001	49.50; 50.51
**3** ^**g**^	**10017.494**	**10106.999**	**10037.184**	**0.959**	**0.004**	**<0.001**	**38.51; 17.57; 43.92**
4	9737.265	9851.181	9762.325	0.972	0.037	<0.001	0.93; 38.81; 42.74; 17.53

^a^AIC, Akaike information criterion; ^b^BIC, Bayesian information criterion; ^c^aBIC, sample-size adjusted Bayesian information criterion; ^d^LMR, Lo–Mendell–Rubin adjusted likelihood ratio test; ^e^P, probability value; ^f^BLRT, bootstrapped likelihood ratio test; ^g^Bold indicates the selected model.

Table 2 presents the average posterior probabilities for the three-profile solution. The average assignment probabilities were 0.985 for low PI (C1), 0.972 for moderate PI (C3) and 0.992 for high PI (C2). These values showed clear separation across the three profiles.

**Table 2:** Average posterior probabilities for the three-profile solution.

Assigned profile	Posterior probability of membership^h^		
	C1 Low PI profile^i^ (*n* = 164; 38.0%)	C2 High PI profile (*n* = 76; 17.6%)	C3 Moderate PI profile (*n* = 192; 44.4%)
C1 Low PI profile (n = 164; 38.0%)	**0.985**	0.000	0.015
C2 High PI profile (n = 76; 17.6%)	0.000	**0.992**	0.008
C3 Moderate PI profile (n = 192; 44.4%)	0.025	0.002	**0.972**

^h^C1, low PI profile; C2, high PI profile; C3, moderate PI profile; ^i^PI, professional identity. Bold values on the diagonal indicate the average posterior probability of membership in the assigned profile.

### Profile characteristics

The three profiles showed a clear graded pattern across the five PI dimensions. C1 included 164 nursing interns (38.0%) and had the lowest scores across the five dimensions. This class was labelled the low PI profile. C2 included 76 nursing interns (17.6%) and had the highest scores across all five dimensions. This class was labelled the high PI profile. C3 included 192 nursing interns (44.4%) and showed middle-level scores across the five dimensions. This class was labelled the moderate PI profile. The LPA model estimated profile proportions were 38.5, 43.9, and 17.6%, which differed slightly from the count-based proportions because classification was based on maximum posterior probability. Figure 1 shows the score patterns of the three profiles across the five PI dimensions.

**Figure 1:**
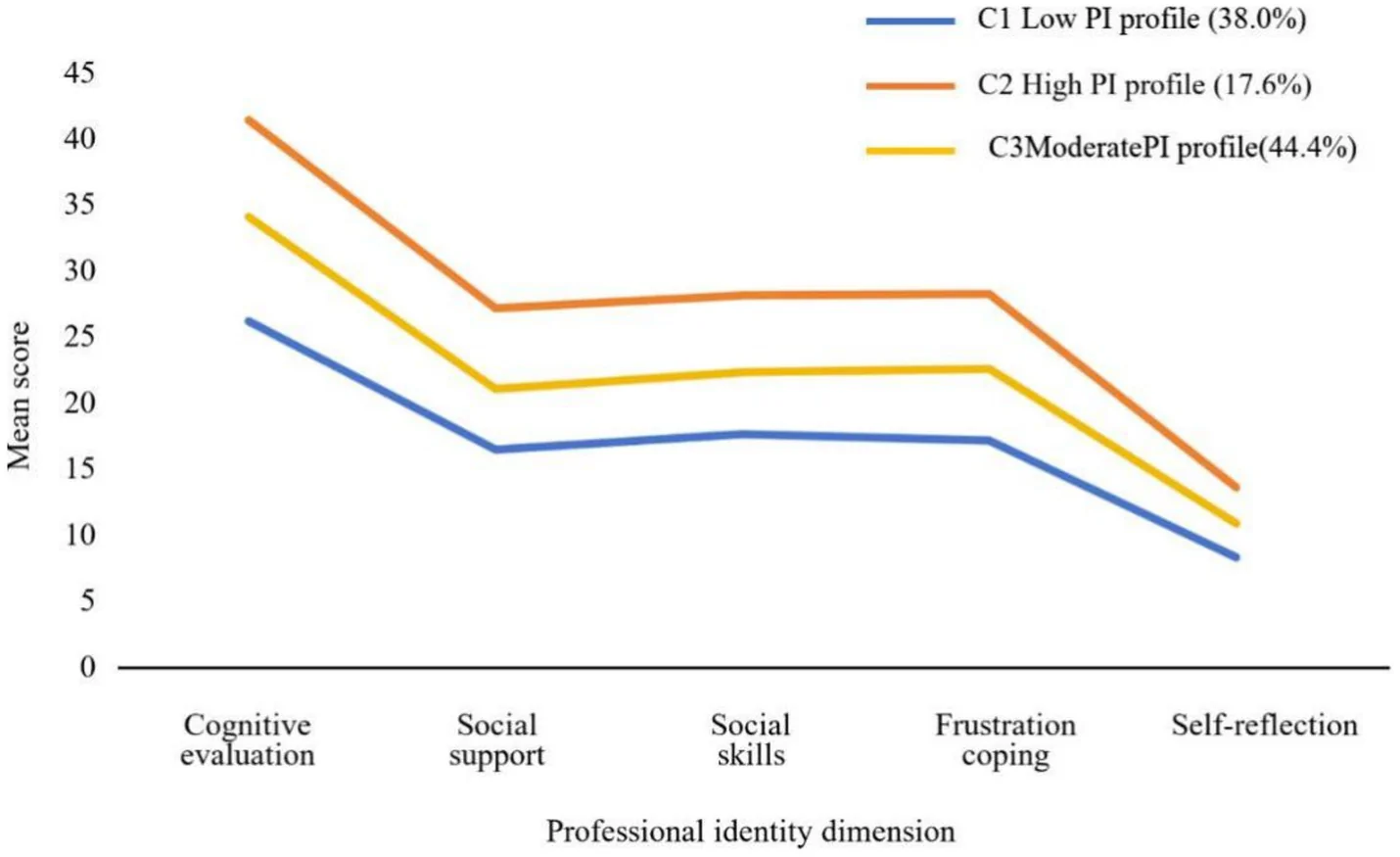
Professional identity dimension scores by latent profile.

### Profile differences in demographic variables and PsyCap

Table 3 shows demographic differences across low PI (C1), moderate PI (C3) and high PI (C2). Sex, age group, educational level and practicum duration were similar across profiles. Failed courses differed across profiles (*p* = 0.045). The low PI profile (C1) contained the largest share of interns with at least three failed courses (4.88%). The moderate PI profile (C3) contained two interns in this category (1.04%). The high PI profile (C2) contained none.

**Table 3:** Demographic characteristics across professional identity profiles.

Characteristic/category^j^	C1 (*n* = 164)	C2 (*n* = 76)	C3 (*n* = 192)^k^	Test statistic	P^l^
Sex				χ^2^ = 1.59^m^	0.452
Male	24 (14.63)	16 (21.05)	34 (17.71)		
Female	140 (85.37)	60 (78.95)	158 (82.29)		
Age group				—	0.075
20–21 years	118 (71.95)	58 (76.32)	122 (63.54)		
22–23 years	42 (25.61)	16 (21.05)	68 (35.42)		
≥24 years	4 (2.44)	2 (2.63)	2 (1.04)		
Educational level				χ^2^ = 3.92	0.141
Bachelor’s degree programme	64 (39.02)	20 (26.32)	72 (37.50)		
Junior college programme	100 (60.98)	56 (73.68)	120 (62.50)		
Number of failed courses				—	0.045
0	132 (80.49)	60 (78.95)	148 (77.08)		
1–2	24 (14.63)	16 (21.05)	42 (21.88)		
≥3	8 (4.88)	0 (0.00)	2 (1.04)		
Cumulative clinical practicum duration				χ^2^ = 5.66	0.226
1–3 months	12 (7.32)	2 (2.63)	10 (5.21)		
4–7 months	72 (43.90)	26 (34.21)	84 (43.75)		
≥8 months	80 (48.78)	48 (63.16)	98 (51.04)		

^j^Values are *n* (%); ^k^C1, low PI profile; C2, high PI profile; C3, moderate PI profile; ^l^P, probability value; ^m^χ^2^, chi-square statistic.

Table 4 shows PsyCap scores across low PI (C1), moderate PI (C3) and high PI (C2). Hope, self-efficacy, resilience and optimism all differed across profiles (all *p* < 0.001). Mean scores for all four PsyCap dimensions were progressively higher across the low, moderate, and high PI profiles.

**Table 4:** Psychological capital scores across professional identity profiles.

PsyCap dimension^n^	Mean (SD)^o^			F	P^p^
	C1 (*n* = 164)	C2 (*n* = 76)	C3 (*n* = 192)		
Hope	23.93 (3.57)	**33.55 (3.02)**	28.11 (3.48)	209.14	<0.001
Self-efficacy	23.40 (4.02)	**33.08 (3.99)**	28.57 (2.85)	212.75	<0.001
Resilience	15.34 (2.71)	**22.26 (2.26)**	18.74 (2.27)	221.43	<0.001
Optimism	11.57 (2.55)	**16.92 (1.75)**	14.25 (1.45)	202.13	<0.001

^n^PsyCap, psychological capital; ^o^C1, low PI profile; C2, high PI profile; C3, moderate PI profile; SD, standard deviation; ^p^P, probability value. Bold values indicate the highest mean score among the three profiles for each psychological capital dimension.

### Factors associated with latent profile membership

Table 5 presents the multinomial logistic regression model with low PI (C1) as the reference group. Membership in moderate PI (C3) was associated with self-efficacy (*OR* = 1.349, *95% CI* = 1.195–1.523, *p* < 0.001) and optimism (*OR* = 1.334, *95% CI* = 1.051–1.693, *p* = 0.018). High PI (C2) was associated with hope (*OR* = 1.344, *95% CI* = 1.103–1.636, *p* = 0.003), self-efficacy (*OR* = 1.412, *95% CI* = 1.221–1.631, *p* < 0.001) and optimism (*OR* = 2.204, *95% CI* = 1.336–3.636, *p* = 0.002). Resilience was not independently associated with profile membership, and failed courses were not interpreted as stable effects because of sparse cells.

**Table 5:** Multinomial logistic regression for professional identity profiles.

Predictor	Contrast^q^	*B^r^*	*SE^s^*	*Z value^t^*	*OR (95% CI)^u^*	P*^v^*
Psychological capital dimensions						
Hope	C2/C1	0.295	0.101	2.938	1.344 (1.103–1.636)	0.003
	C3/C1	−0.083	0.066	−1.256	0.920 (0.808–1.048)	0.209
Self-efficacy	C2/C1	0.345	0.074	4.666	1.412 (1.221–1.631)	<0.001
	C3/C1	0.299	0.062	4.834	1.349 (1.195–1.523)	<0.001
Resilience	C2/C1	0.273	0.201	1.356	1.313 (0.886–1.948)	0.175
	C3/C1	0.152	0.104	1.463	1.164 (0.950–1.428)	0.143
Optimism	C2/C1	0.790	0.255	3.095	2.204 (1.336–3.636)	0.002
	C3/C1	0.288	0.122	2.371	1.334 (1.051–1.693)	0.018
Number of failed courses during school^w^						
1–2 courses	C2/C1	−0.114	0.598	−0.190	0.892 (0.276–2.884)	0.849
	C3/C1	0.329	0.383	0.858	1.390 (0.655–2.946)	0.391
≥3 courses	C2/C1	−7.080	0.000	−180965.590	0.001 (0.001–0.001)	<0.001
	C3/C1	0.085	0.988	0.086	1.089 (0.157–7.548)	0.931

^q^C2/C1, High vs low PI profile; C3/C1, Moderate vs low PI profile; PI, professional identity; ^r^B, regression coefficient; ^s^SE, standard error; ^t^Z value, B divided by SE; ^u^OR, odds ratio; CI, confidence interval; ^v^P, probability value; ^w^0 courses was the reference category.

## Discussion

### Principal findings

Nursing interns are often treated as a relatively homogeneous group because they share a similar training stage. However, the low, moderate, and high PI profiles identified in this study suggest that meaningful heterogeneity exists even among interns at the same practicum stage. The profiles differed across professional cognitive evaluation, professional social support, professional social skills, professional frustration coping, and professional self-reflection, indicating that PI during practicum is linked with how interns understand the nursing role, experience support, manage difficulty, and reflect on their future career (Gong et al., 2025; Tu et al., 2024). It should also be noted that the three profiles followed a largely ordered pattern across all five PI dimensions, suggesting that they mainly represent graded levels of PI rather than sharply different qualitative configurations. Even so, the person-centered approach remains useful because it identifies empirically derived subgroups with different overall levels of identity strength, which may help educators prioritize and tailor the intensity of practicum support.

In addition, PsyCap was associated with differences in PI profile membership among nursing interns. Higher self-efficacy and optimism were associated with membership in both the moderate and high PI profiles, whereas higher hope was associated only with the high PI profile. These findings are broadly consistent with Psychological Capital Theory (Luthans et al., 2006; Feng et al., 2024). Their temporal ordering remains open, and reciprocal relationships are also plausible. For example, stronger PI may be related to greater self-efficacy, optimism, and hope.

### PI profiles in clinical practicum

The three PI profiles identified in this study can be interpreted through five dimensions used to identify them (Liu et al., 2011), namely role understanding, clinical belonging, teamwork, adaptation to difficulty, and reflection on future career identity. Professional cognitive evaluation reflects how interns understand nursing work and its value (Goodolf and Godfrey, 2021; Joseph et al., 2023), whereas professional social support, social skills, frustration coping, and self-reflection capture how they experience recognition, participate in teamwork and patient care, respond to clinical stress, and connect practicum experiences with their future professional identity.

Viewed together, these findings suggest that the three profiles represent professional role integration across these domains, rather than isolated differences in a single dimension. The low PI profile may indicate relatively weaker professional grounding, the moderate PI profile may reflect an intermediate and potentially more malleable stage of role acceptance, the high PI profile may indicate a stronger connection between clinical learning and future professional self. Mao et al. (2021) described PI as a process shaped by personal, educational, and professional factors. The present findings indicate that interns at the same clinical stage may differ in overall PI strength. This pattern may help educators consider different levels of practicum support.

### Implications of PI patterns for clinical practicum support

Previous person-centered studies have shown that nursing students differ in how they respond to clinical learning, stress, and professional development. Li et al. (2023) linked PI profiles with stress and coping during clinical practicum, suggesting that real care settings may make differences in PI more visible. Our study adds a more practice-oriented perspective by showing that higher self-efficacy and optimism were associated with membership in the moderate and high PI profiles, whereas higher hope was associated with membership in the high PI profile, compared with the low PI reference profile. These PsyCap dimensions warrant prospective evaluation as candidate intervention targets.

Zhou et al. (2023) found that PI differed across self-directed learning profiles, highlighting the close relationship between PI and students’ engagement with learning tasks. Our study extends this evidence into clinical practicum by identifying PI profiles among nursing interns and linking these profiles with modifiable psychological resources. Wu et al. (2024) showed that resilience supported PI through personality-related pathways. In our study, resilience differed across profiles but was not a stable predictor after other PsyCap dimensions were considered. The association between resilience and PI may vary with clinical stress, which could be examined in future studies.

### PsyCap in the clinical learning environment

In clinical practicum, the observed associations between PsyCap and PI may reflect differences in how interns interpret feedback, patient contact, teamwork, and pressure (Luthans et al., 2006; Gong et al., 2025). Practicum requires interns to learn procedures, communicate with patients, accept supervision, and manage emotional demands. These experiences may be interpreted either as opportunities for growth or as sources of uncontrolled stress, and PsyCap may influence this process of interpretation and adaptation.

Among the PsyCap dimensions, self-efficacy showed the most consistent association. This pattern may reflect a connection between clinical confidence and professional self-concept during the transition to practice (Abusubhiah et al., 2023). Repeated mastery experiences may arise from patient observation, communication, documentation, and teamwork, while clear preceptor feedback may help interns translate these experiences into a stronger sense of professional belonging (Zhang et al., 2022). Feng et al. (2024) also reported a positive association between PsyCap and PI among undergraduate nursing students.

In addition, optimism may be linked to interns framing clinical difficulty as manageable. Hope was related to daily practicum tasks and longer-term career goals. Gong et al. (2025) found that PsyCap, stress, the micro-learning environment, and PI were interrelated among nursing interns. This supports the view that psychological resources operate within daily learning conditions.

From this perspective, the clinical learning environment provides the context in which interns encounter preceptor feedback, patient contact, peer interaction, department culture, workload, and opportunities for reflection. Gao et al. (2022) found that career self-efficacy mediated the relationship between clinical learning environment and PI in nursing students. In the present study, practicum duration was not associated with profile membership, indicating that time in placement should not be treated as a proxy for the quality of the clinical learning environment.

### Implications for nursing education and retention

The findings support profile-based practicum support. Interns in the low PI profile may need structured orientation, closer preceptor contact, and guided reflection on the value of nursing work. Interns in the moderate PI profile may benefit from progressive task assignment, competence feedback, and career planning. Interns in the high PI profile may benefit from advanced learning tasks, peer mentoring, and quality improvement activities.

Self-efficacy can be strengthened through mastery experiences, simulation preparation, and skills feedback. Optimism can be supported through reflective discussion and constructive interpretation of setbacks. Hope can be strengthened through goal setting and visible practicum pathways. These strategies connect psychological support with clinical teaching and workforce preparation. Table 6 summarizes profile-informed educational needs and potential practicum interventions.

**Table 6:** Profile-informed educational considerations for clinical practicum support.

PI^x^ profile	Main educational need	Potential intervention
Low PI (C1)	Role clarity, clinical belonging, and professional confidence	Structured orientation, close preceptor supervision, mastery feedback, and guided reflection
Moderate PI (C3)	Consolidation of role integration and confidence	Progressive task assignment, competence feedback, and career planning
High PI (C2)	Continued development and meaningful professional engagement	Advanced learning tasks, peer mentoring, and quality improvement activities

^x^PI, professional identity.

PI also has relevance for workforce retention. Nursing interns are part of the future nursing workforce. Strong PI may support career commitment and willingness to remain in nursing. Kristoffersen (2021) have linked PI with decisions to remain in nursing. Early identification of low PI may guide timely transition support.

### Strengths and limitations

This study has several strengths. It focused on nursing interns during clinical practicum, a stage when students begin to translate classroom learning into clinical role experience. It used LPA to identify PI heterogeneity directly, which provides more practical information than an average PI score. The study also combined Professional Identity in Nursing with Psychological Capital Theory, allowing the PI profiles to be interpreted through both professional development and positive psychological resources. These strengths make the findings useful for designing profile-based support during clinical practicum.

Several limitations should be considered. The cross-sectional design limits interpretation of change over time. The observed pattern may reflect reciprocal links between PsyCap and PI. Higher PI scores may also occur alongside higher self-efficacy, optimism, and hope. Longitudinal studies could clarify how these relationships unfold over time. The sample came from two tertiary general hospitals in Chengdu, which may limit transferability to other regions, hospital levels, and educational systems. The online questionnaire format may introduce response bias. The study also lacked measures of preceptor support, department culture, workload, workplace bullying, and patient care pressure. These factors may explain further differences in PI profile membership. In addition, because the identified profiles were largely distinguished by overall score level, they should be interpreted cautiously as ordered subgroups within a professional identity continuum rather than as fully distinct identity types.

### Future research

Future longitudinal studies should examine reciprocal changes in PsyCap and PI profile membership before, during, and after clinical practicum. Multicentre studies across different hospital levels and regions can test the stability of the three-profile structure. Intervention studies could evaluate whether mastery-based feedback, guided reflection, goal-focused mentoring, and structured preceptor support are associated with changes in PI scores or transitions between profiles.

Future studies could examine whether preceptor support, workload, department climate and stressful events are related to changes in PI and PsyCap over time. Qualitative interviews could explore how interns from different profiles interpret clinical experiences and their developing professional identity. These next steps can help educators identify which interns need closer supervision, confidence-building feedback, or goal-focused mentoring during clinical practicum.

## Conclusion

This study identified low, moderate, and high PI profiles among nursing interns during clinical practicum. The ordered pattern reflected differences in overall PI strength across role understanding, clinical belonging, teamwork, coping and reflection. Self-efficacy and optimism were associated with membership in the moderate and high PI profiles, whereas hope was associated only with the high PI profile. These associations may inform the design and future evaluation of confidence-building feedback, goal-focused mentoring and guided reflection. The cross-sectional design leaves temporal ordering open, and the two-hospital sample may limit transferability. Future longitudinal, multicentre, and intervention studies can test whether these patterns remain stable and whether targeted practicum support helps interns build clearer professional role meaning.

## Data Availability

The raw data supporting the conclusions of this article will be made available by the authors, without undue reservation.
